# Editorial Comment: Ascending testis: A congenital predetermined condition

**DOI:** 10.1590/S1677-5538.IBJU.2022.01.06

**Published:** 2021-12-21

**Authors:** Natasha T. Logsdon, Luciano A. Favorito

**Affiliations:** 1 Universidade do Estado do Rio de Janeiro Unidade de Pesquisa Urogenital Rio de Janeiro RJ Brasil Unidade de Pesquisa Urogenital - Universidade do Estado do Rio de Janeiro - Uerj, Rio de Janeiro, RJ, Brasil

Nasib Alchoikani 1, Khaled Ashour 2

J Pediatr Urol. 2021 Apr;17(2):192.e1-192.e3.

## COMMENT

In this interesting paper the authors studied the intra-operative findings of patients underwent orchidopexy for ascending testes and concluded that the patients with ascending testes seem to share similar intra-operative findings with patients who have true undescended testes. The authors show the presence of abnormal attachment of the gubernaculum and epididymal anomalies in the groups studied. The gubernaculum seems to be the most important anatomical structure in the testicular migration process, by means of contraction and shortening, thus imposing traction strength on the testis and facilitates the transition of the testis by through the inguinal canal (1). Undescended testis can be associated with several anatomical anomalies but the abnormalities in gubernaculum insertions and the presence of epididymal anomalies of disjunction and atresia are among the most common (2). During the surgery of undescended testis, specially in those with abdominal position, the knowledge of the testicular vascularization are very useful to preserver the testis (3,4). This paper indicates that that ascending testis should be considered a congenital predetermined condition and during the child growth stay in a higher position.
